# Measurement of Health Program Equity Made Easier: Validation of a Simplified Asset Index Using Program Data From Honduras and Senegal

**DOI:** 10.9745/GHSP-D-15-00385

**Published:** 2016-03-25

**Authors:** Alex Ergo, Julie Ritter, Davidson R Gwatkin, Nancy Binkin

**Affiliations:** a Broad Branch Associates, Washington, DC, USA; b University of Tennessee Health Science Center, Department of Preventive Medicine, Memphis, TN, USA; c Results for Development Institute, Washington, DC, USA; d UC San Diego School of Medicine, Department of Family Medicine and Public Health, San Diego, CA, USA

## Abstract

Equitable access to programs and health services is essential to achieving national and international health goals, but it is rarely assessed because of perceived measurement challenges. One of these challenges concerns the complexities of collecting the data needed to construct asset or wealth indices, which can involve asking as many as 40 survey questions, many with multiple responses. To determine whether the number of variables and questions could be reduced to a level low enough for more routine inclusion in evaluations and research without compromising programmatic conclusions, we used data from a program evaluation in Honduras that compared a pro-poor intervention with government clinic performance as well as data from a results-based financing project in Senegal. In both, the full Demographic and Health Survey (DHS) asset questionnaires had been used as part of the evaluations. Using the full DHS results as the “gold standard,” we examined the effect of retaining successively smaller numbers of variables on the classification of the program clients in wealth quintiles. Principal components analysis was used to identify those variables in each country that demonstrated minimal absolute factor loading values for 8 different thresholds, ranging from 0.05 to 0.70. Cohen’s kappa statistic was used to assess correlation. We found that the 111 asset variables and 41 questions in the Honduras DHS could be reduced to 9 variables, captured by only 8 survey questions (kappa statistic, 0.634), without substantially altering the wealth quintile distributions for either the pro-poor program or the government clinics or changing the resulting policy conclusions. In Senegal, the 103 asset variables and 36 questions could be reduced to 32 variables and 20 questions (kappa statistic, 0.882) while maintaining a consistent mix of users in each of the 2 lowest quintiles. Less than 60% of the asset variables in the 2 countries’ full DHS asset indices overlapped, and in none of the 8 simplified asset index iterations did this proportion exceed 50%. We conclude that substantially reducing the number of variables and questions used to assess equity is feasible, producing valid results and providing a less burdensome way for program implementers or researchers to evaluate whether their interventions are pro-poor. Developing a standardized, simplified asset questionnaire that could be used across countries may prove difficult, however, given that the variables that contribute the most to the asset index are largely country-specific.

## Abstract (precis)

Piggy-backing on an existing representative household survey that includes an asset index, it is possible to assess the socioeconomic distribution of program beneficiaries at low cost. The typically large number of questions used to construct the asset index, however, deters many implementers from adopting this approach. This study demonstrates that the number of questions can be significantly reduced to a subset that takes only a few minutes to administer without substantially altering findings or policy recommendations. The relevant subset is country-specific and thus necessitates tailored country questionnaires.

## INTRODUCTION

Considerable efforts have been made over the past 2 decades to raise awareness among public health professionals of the importance of incorporating an equity perspective into health-related policies and interventions in low- and middle-income countries (LMICs). A number of publications have greatly contributed to these efforts by documenting prevailing socioeconomic inequalities in health,^1^ showing what works and what does not to improve equity,^2^ and providing useful tools and methods for assessing equity.^3^^-^^5^

These efforts have been accompanied by an increased interest in the use of a so-called asset index (or wealth index) to measure socioeconomic position in LMICs. An asset index combines responses to survey questions regarding household asset ownership, housing characteristics, and access to basic services such as water and sanitation. Principal components analysis (PCA) is commonly used to calculate a weight for each variable generated from the responses to those questions.^6^ An index is then calculated for each household by adding up the weighted responses. Each household is thus given an asset index value. All the households in the survey are subsequently sorted based on the value of their asset index and the sample is divided into socioeconomic groups of equal size. Most commonly, 5 groups are created, which are known as asset quintiles or wealth quintiles.^7^ The use of an asset index is particularly attractive for the analysis of data from household surveys such as the Demographic and Health Surveys (DHS), the Multiple Indicator Cluster Surveys (MICS), or the Reproductive Health Surveys (RHS) because of difficulties in many LMICs in obtaining accurate information regarding household income or consumption expenditure.

An asset index can be used for a wide range of equity analyses. It can, for example, be used to assess the socioeconomic profile of users of a specific health service^8^ by asking a small random sample of service users the same questions as those used for the construction of an asset index in an existing reference survey such as the DHS. The asset index is then constructed for each service user, using the same weights as those used to construct the asset index in the reference survey. The asset indices of service users can then be compared with those of people in the country as a whole, i.e., those in the reference survey, which permits estimation of the proportion of service users falling into each of the national asset quintiles. Patterns can then be examined to assess whether users are evenly spread across quintiles; whether they are more represented in the lower quintiles, in which case the program or intervention is pro-poor; or conversely whether relatively more service users fall into the higher quintiles, indicating that the program or intervention favors the better-off.

This method of assessing equity in use of health services is attractive for 2 reasons: it is relatively low cost and it is easy to apply. More particularly, it has the following advantages:

- The socioeconomic profile of service users can be compared with national asset indices that are already calculated, thus considerably simplifying the calculations needed to analyze the survey of intervention beneficiaries.
- Country-specific asset questions developed by those who conducted the reference survey can simply be added to a planned survey, including household surveys or exit surveys of facility users.
- The sample size required is relatively small since there is no need to *create* new asset quintiles from the sample of service users. The analysis in essence *borrows* information from an existing household survey, in which a representative sample of the national population was already divided into national wealth quintiles.

Despite these advantages, many implementers who wish to assess whether their interventions have reached the poor may still perceive the approach as being too burdensome, especially since the construction of the asset index in a typical reference survey, such as a DHS, can involve as many as 40 questions, many with multiple responses. They may be reluctant to add that many extra questions to a survey that is primarily designed to monitor overall progress or achievements relating to the service or intervention of interest.

Construction of the standard asset index can involve as many as 40 survey questions, making it burdensome for program implementers to use.

Using data from previously conducted studies in Honduras and Senegal, this paper examines the extent to which a reduction in the number of variables used to construct the asset index, and consequently in the number of questions to be asked of service users, affects the results of such equity analysis. Most importantly, the paper assesses the extent to which such reduction alters the resulting socioeconomic profile of service users and the policy recommendations derived from the findings.

## METHODS

Our analysis was based on data from 2 countries: Honduras and Senegal. In each of these countries, a suitable example of the full equity analysis described in the preceding section was available. Both examples used a DHS as reference survey.

### Project Implemented by Child Fund International in Honduras

From October 1, 2009, to September 30, 2013, Child Fund International (CFI) implemented a community-based maternal, neonatal, and child health project in Francisco Morazán Sur, Honduras. The goal of the project was to decrease maternal, neonatal, infant, and under-5 child mortality in the project area through 3 community-based health interventions:

1. Standardizing the role of communities in increasing institutional deliveries and strengthening community-based obstetric and neonatal care within a national decentralization strategy.
2. Creating self-sustaining community-based health units (UCOS), which integrate vertical Ministry of Health (MOH) maternal, neonatal and child health programs and various cadres of community volunteers. UCOS are small, freestanding structures located in selected communities, equipped with essential drugs, basic equipment, and health education materials. Community volunteers offer care, attention, and education to persons in need, with an emphasis on women, infants, and children. They are self-sustaining financially, managed by the community, supervised by the MOH, and given technical and logistical support by Child Fund Honduras. UCOS sustainability depends upon a functioning revolving drug fund.
3. Adapting and implementing community-based continuous quality improvement systems for the UCOS.

The UCOS strategy was developed to better reach the most underserved populations, which tend to be families of low socioeconomic status. To determine whether the UCOS were reaching a poorer population than MOH facilities (referred to as CESAMO), CFI conducted exit surveys of 334 UCOS clients and 143 clients of CESAMO facilities, after obtaining written informed consent. The full set of questions used to construct an asset index in the 2011–2012 Honduras DHS was added to the CFI questionnaire, and asset indices were calculated for all respondents using the DHS weights. Respondents were then assigned to a national asset quintile based on the value of their asset index, and a socioeconomic profile of service users was constructed for the 2 types of facilities. The exit surveys complemented assessments of health coverage and costs. The MOH was interested in reviewing information from all 3 assessments to determine if the UCOS strategy should be adopted as national policy.

### Project Implemented by the Ministry of Health and Social Action in Senegal

In 2012, the Ministry of Health and Social Action (MoHSA) of Senegal implemented a results-based financing (RBF) pilot project in 2 regions of the country: Kaffrine and Kolda. In this pilot, financial incentives were provided to health centers, district hospitals, and district health management teams, conditional on meeting predefined targets on a set of key health service utilization indicators. Financial incentives also reflected quality of care, which was assessed using a quality checklist.

Equity was not explicitly taken into consideration in the design of the pilot. MoHSA was well aware, however, of the risk that RBF might encourage health facilities to focus on populations that are easier to reach—which tend to be better off—in order to meet the targets and that this would lead to increased socioeconomic inequalities in health. It was therefore important to monitor the equity effect of the pilot. This monitoring was incorporated into the verification function of the RBF model.

As part of the verification process, facility registers were reviewed, and during the review process the verification team also extracted information on a random sample of patients. A contracted community-based organization was then tasked with visiting the households of the selected patients and, after obtaining informed consent, to verify the data extracted from the facility’s registers. Interviewed service users from Kaffrine and Kolda districts (N = 1,423) were also asked about expenses engendered and perceived quality. In addition, the questionnaire included all the questions and response options used in the 2010–2011 Senegal DHS to construct an asset index. The weights applied by DHS were then used to calculate a comparable asset index value for each respondent’s household, which could then be used to construct the socioeconomic profile of service users.

### Development of a “Simplified” Asset Index

Both country studies relied on the “full” asset index, as constructed in the reference DHS, constituting the ideal starting point for our analysis. Results obtained in each of these applications became the “gold standard” with which we compared results from analyses based on simplified asset indices—that is, asset indices constructed using a shorter list of variables.

Simplified asset indices were validated against program data from Honduras and Senegal, with the standard DHS wealth index in each country serving as the reference.

In both Honduras and Senegal, DHS produces separate asset indices for urban and rural households, using PCA. The urban and rural household asset indices are then combined into a national asset index, using a third common PCA coupled with a regression procedure developed by the DHS secretariat.^9^^,^^10^ The resulting national asset index is used to construct the national asset quintiles. In our study, we followed the same procedure to develop a national asset index for each study country that is identical to that included in the DHS dataset.

We then examined the individual variables contained in each country’s index. To begin, we sorted the variables in ascending order of their relative importance, as captured by the absolute value of factor loadings.

After sorting the variables, we identified and dropped those that contributed least to the DHS asset index’s total value, i.e., those at the top of the sorted lists. Rather than dropping variables one by one, we dropped the variables in groups by gradually increasing the threshold for the minimum acceptable factor loading. More precisely, we first narrowed down the list of variables by only accepting variables with an absolute value of factor loading greater than 0.05; we subsequently increased the threshold to 0.10, 0.20, and so forth up to 0.70, resulting in a total of 8 iterations. We stopped at a threshold of 0.70 because in both countries the correlation to the original asset index at that threshold was visibly far off and neither country had more than 3 variables that would meet a higher threshold. Variables needed to meet the threshold for the absolute value of factor loading in at least one of the PCAs (urban, rural, or common) in order to be retained within each respective iteration. To maintain consistency, we based variable selection in each iteration on the same sorted list derived from the full asset index. The asset index calculation procedure described above, however, was repeated on each shortened list of variables to generate new weights and quintile cut-off points. PCA was conducted using SPSS factor analysis procedure (SPSS Statistics Version 22), extracting only one factor.

### Socioeconomic Profile of Honduras and Senegal Service Users

To construct the socioeconomic profile of service users in the Honduras and Senegal studies, we used the procedure described earlier for the original DHS asset index to calculate a national asset index value for each participant. We then applied the original DHS asset quintile cut-off points to classify the participants in the 2 Honduras programs and the single Senegalese program into national asset index quintiles. These steps were then repeated for each of the 8 simplified asset index iterations. Results obtained for each of the iterations were compared against the results based on the original DHS asset index, which was considered to be the gold standard for these analyses.

### Evaluation of the “Parsimonious” Asset Index Iterations

We used Cohen’s kappa statistic for 2 different purposes. First, we used it to compare the composition of the DHS asset quintiles, which represent the general population: in comparison with the quintiles based on the “full” asset index, how did the composition change in each iteration, i.e., what proportion of households moved in and out of each quintile? Second, we used Cohen’s kappa statistic to compare the socioeconomic profiles of service users: in comparison with the socioeconomic profile obtained in the original analysis, how did the profile change in each of the 8 iterations based on a simplified asset index? This test, which is considered to be a more robust measure than a simple percent agreement calculation since it takes into account the agreement occurring by chance, produces values ranging from -1 to 1. Correlations <0 are considered to have poor strength of agreement, with 0–0.20 considered as slight, 0.21–0.40 as fair, 0.41–0.60 as moderate, 0.61–0.80 as substantial, and 0.81–1.0 as almost perfect agreement.^11^

In order to assess changes in the socioeconomic profile of service users, we also calculated the difference in the percentage of respondents in each asset quintile compared with that obtained in the analysis based on the original DHS asset index. For summary purposes, we present the highest percentage point change across the 5 quintiles for each of the iterations.

To examine the extent to which the use of a simplified asset index would result in similar conclusions from a policy standpoint, we used the Honduras data in which the purpose of the evaluation had been to compare the ability of the UCOS service delivery model to enroll a higher percentage of the poor than the CESAMO model. We compared, for each iteration, the percentage of clients in each asset quintile for the 2 delivery models to determine if the patterns observed in the original analysis, which was based on the full DHS asset index, persisted as the number of variables included in the asset index was decreased. For purposes of this analysis, we used 4 iterations (numbers 1, 2, 4, and 7 described fully in the Results section), which involve 111 (full asset index), 74, 36, and 9 variables, respectively.

While assessing the agreement between asset quintile assignments is essential in any attempts to reduce the number of asset variables, a second essential component in developing a more parsimonious asset index is evaluating the extent to which it simplifies and shortens data collection. In the asset index calculations used by DHS and others, multiple choice responses to questions relating to the source of drinking water, the type of fuel used for cooking, or the material used for wall, roofing, or floors, are each coded as binary variables, such that a single question may generate many variables. For this reason, we examined not only the variables in the simplified indices but also the number of questions that would need to be asked on a questionnaire to generate the retained variables.

Our final analysis consisted of a comparison of the lists of asset variables retained in each iteration between the 2 countries. The purpose was to assess the extent to which a single set of asset variables could be used across the 2 countries.

## RESULTS

Table 1 presents the principal results for Honduras, for each of 8 iterations, while Table 2 presents the results for Senegal. As shown in columns (c) and (d) of the tables, the number of required questions and variables for each country declined as the factor loading cut-offs were raised, although the number of questions that would need to be retained did not decline as rapidly as the number of variables.

**TABLE 1:** Honduras Results for Full Asset Index and 8 Simplified Iterations

Iteration	Inclusion Criteria (absolute value of the factor loading)	No. of Questions	No. of Variables	Changes in DHS Quintile Composition Kappa Statistic (N = 21,362)	Changes in Socioeconomic Profile		
					Max. Absolute Percentage Point Change *(in which quintile)*	Kappa Statistic	
						UCOS (n = 334)	CESAMO (n = 143)
(a)	(b)	(c)	(d)	(e)	(f)	(g)	(h)
Full asset index (reference)	All variables included	41	111	1.000	NA	1.000	1.000
1	>0.05	35	86	0.993	3% *(CESAMO Q2&3)*	0.972	0.957
2	>0.10	33	74	0.986	2% *(CESAMO Q3)*	0.966	0.936
3	>0.20	25	48	0.927	6% *(CESAMO Q3)*	0.898	0.780
4	>0.30	19	36	0.877	4% *(CESAMO Q2)*	0.829	0.734
5	>0.40	15	24	0.799	8% *(CESAMO Q2)*	0.778	0.652
6	>0.50	13	17	0.724	11% *(CESAMO Q4)*	0.683	0.471
7	>0.60	8	9	0.634	8% *(CESAMO Q2)*	0.476	0.422
8	>0.70	2	2	0.209	91% *(UCOS Q3)*	-0.023	0.019

Abbreviations: CESAMO, Centro de Salud con Médico y Odontólogo (Ministry of Health clinics); DHS, Demographic and Health Survey; UCOS, Unidades Comunitarios (community-based health units).

**TABLE 2:** Senegal Results for Full Asset Index and 8 Simplified Iterations

Iteration	Inclusion Criteria (absolute value of the factor loading)	No. of Questions	No. of Variables	Changes in DHS Quintile Composition Kappa Statistic (N = 7,902)	Changes in Socioeconomic Profile	
					Maximum Absolute Percentage Point Change *(in which quintile)*	Kappa Statistic (N = 1,423)
(a)	(b)	(c)	(d)	(e)	(f)	(g)
Full asset index (reference)	All variables included	36	103	1.000	NA	1.000
1	>0.05	34	82	0.997	1% *(Q1&2)*	0.925
2	>0.10	32	63	0.969	1% *(Q1&2)*	0.950
3	>0.20	24	40	0.920	4% *(Q1&2)*	0.867
4	>0.30	20	32	0.882	8% *(Q1)*	0.751
5	>0.40	14	21	0.814	7% *(Q2)*	0.746
6	>0.50	11	16	0.779	8% *(Q1&2)*	0.713
7	>0.60	9	10	0.675	12% *(Q1&2)*	0.553
8	>0.70	3	4	0.310	42% *(Q2)*	0.231

The kappa statistics in column (e) show the level of agreement in the DHS data between asset quintiles based on the original DHS asset index and those based on each simplified asset index iteration. For Honduras, there was *almost perfect* agreement all the way down to iteration 4 (and almost to iteration 5; 36 to 24 variables) (Table 1), and for Senegal, to iteration 5 (21 variables) (Table 2). Thus, for these first 4 to 5 iterations, changes in the quintile composition were relatively minor, and reducing the number of asset variables, from 111 to 36 in the case of Honduras and from 103 to 21 in the case of Senegal, had limited effect on the DHS asset quintile composition. For both countries, the level of agreement remained *substantial* (i.e., with a kappa value greater than 0.61) in all but the last iteration, suggesting that the number of asset variables could potentially be further reduced down to 9 for Honduras and to 10 for Senegal.

The number of asset variables in the wealth index could potentially be reduced from 111 to 9 in Honduras, and from 103 to 10 in Senegal, while maintaining substantial agreement with the standard wealth index.

Columns (f), (g), and (h) in Table 1, and columns (f) and (g) in Table 2, summarize the findings relating to changes in the socioeconomic profile of the service users in each country’s project. Column (f) in both tables indicates in which quintile(s) the largest change was observed, and for Honduras (Table 1), in which study group (UCOS or CESAMO). The maximum change remained below 10 percentage points for iterations 1 to 5 in Honduras (111 to 24 variables) and for iterations 1 to 6 in Senegal (103 to 16 variables). In Honduras, the largest changes tended to occur in the socioeconomic profile of CESAMO users. In neither of the countries did the maximum change exceed 12 percentage points until the very last iteration. The kappa statistics displayed in the remaining column(s) were systematically lower than those relating to the DHS asset quintile composition in column (e), although the correlations remained in the “substantial” or higher category until the fifth iteration in Honduras (24 variables) and the sixth iteration in Senegal (16 variables).

**FIGURE 1:**
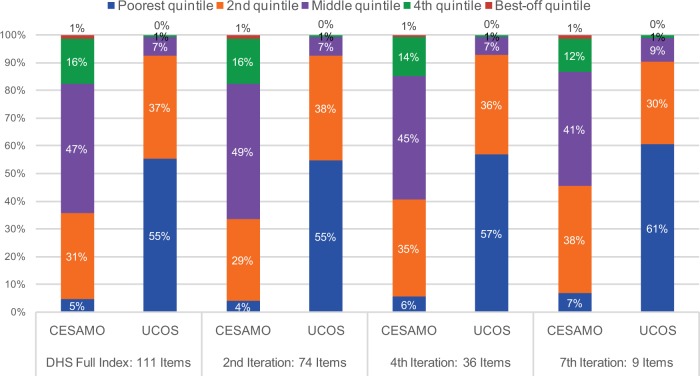
Socioeconomic Profile of Service Users Based on the DHS Full Asset Index and for 8 Simplified Iterations, Using Data From (a) Honduras UCOS, (b) Honduras CESAMO, and (c) Senegal Abbreviations: CESAMO, Centro de Salud con Médico y Odontólogo (Ministry of Health clinics); DHS, Demographic and Health Survey; UCOS, Unidades Comunitarios (community-based health units).

Figure 1 shows changes in the socioeconomic profile of service users as the number of variables used to construct the asset index decreases for Honduras (1a and 1b) and for Senegal (1c). In each graph, the socioeconomic profile obtained in the original study, which used the full asset index and which was defined as the gold standard for this exercise, is displayed on the left. In all 3 cases, we see that despite slight variations in the proportion of users in each quintile, the socioeconomic profile was not dramatically affected by a simplification of the asset index, at least up to a certain point, namely until the variable number decreased below 9 items for Honduras and below 10 items for Senegal. The Senegal data and the Honduras UCOS data suggest a pro-poor policy in which the percentages of users in the lowest 2 quintiles vastly exceeded the expected 40% in these categories. However, for Senegal, the relative mix of users from the first and second quintiles changed, whereby from the fifth iteration onward (21 or fewer variables), the lowest 2 quintiles are almost equally represented among service users.

The socioeconomic profile of users generally was not dramatically affected by simplification of the asset index.

**FIGURE 2:**
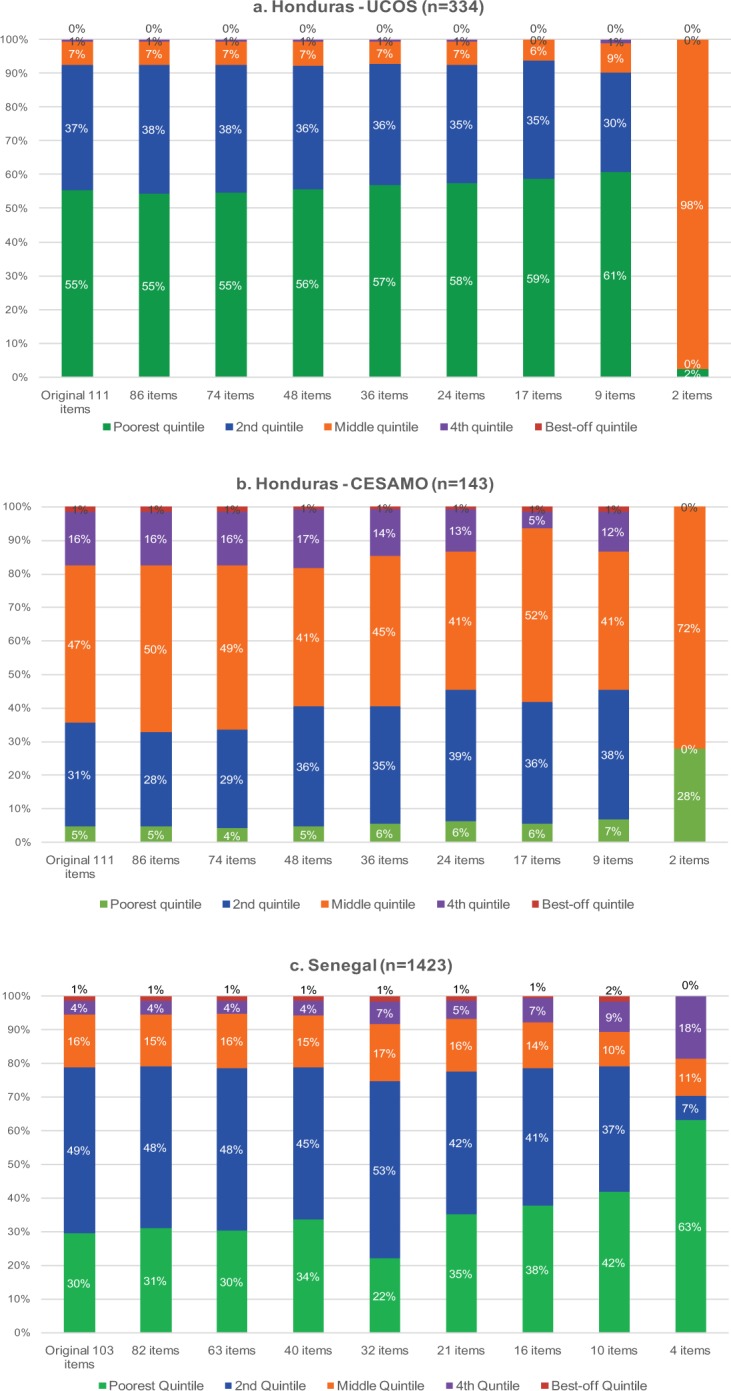
Socioeconomic Profile of Service Users, by Provider Type, Based on the DHS Full Asset Index and for Selected Iterations, Using Data From Honduras Abbreviation: DHS, Demographic and Health Survey.

To examine the extent to which the use of a simplified asset index would result in similar conclusions from a policy standpoint, Figure 2 presents a comparison of the 2 Honduras user populations under 4 scenarios: all 111 variables (used as the gold standard), 74 variables, 36 variables, and 9 variables. These data suggest that the main messages would remain the same, even for the iteration in which the asset index was constructed using only 9 asset variables: (1) UCOS is pro-poor, and (2) it is more pro-poor than CESAMO.

The number of variables required to construct the asset index can be reduced considerably without leading to different policy recommendations.

In terms of the possibility of using a single asset index across countries, Table 3 shows, for each iteration, how much overlap occurred between the lists of asset variables in each of the 2 countries. Of note, the list of asset variables included in the original full asset index, which was part of the DHS dataset, differed considerably between the 2 countries, with the 2 lists having less than 60% of asset variables in common. In none of the iterations did this proportion exceed 50%.

**TABLE 3:** Comparison of Variables Included in Honduras and Senegal Iterations

Iteration	Inclusion Criteria (absolute value of the factor loading)	No. of Variables Included		
		Honduras (Total)	Overlapping	Senegal (Total)
Full asset index (reference)	All variables included	111	57	103
1	>0.05	86	39	82
2	>0.10	74	29	63
3	>0.20	48	18	40
4	>0.30	36	13	32
5	>0.40	24	9	21
6	>0.50	17	5	16
7	>0.60	9	3	10
8	>0.70	2	0	4

## DISCUSSION

Although equity in health service utilization is considered of great importance, it is rarely evaluated at the level of programs or interventions because of concerns about administering a long and complex questionnaire. In this study, we sought to evaluate the scope for simplifying the asset index by reducing the number of variables included in its calculation. Using 2 concrete examples from 2 different countries where a recent household survey with an asset index—in this case a DHS—was available, we assessed the extent to which a simplification of the asset index affects the main message and possible associated policy recommendations. These 2 examples clearly demonstrate that the number of variables and the number of questions required to construct the asset index can be reduced considerably without substantially altering the main findings, and without leading to different policy recommendations.

The extent to which the list of variables and questions can be reduced varied between study sites. In the Honduras example, the 111 asset variables derived from 41 questions could be reduced to as few as 9 variables, captured by only 8 questions, without altering the main conclusions. In the Senegal example, in which the initial questionnaire contained 103 asset variables and 36 questions, the possible reduction appeared to be more modest, with findings, especially in terms of the percentage of clients in each of the 2 lowest quintiles, remaining consistent when the number of variables was reduced down to 32 and the number of questions to 20. One possible explanation for this difference between the 2 country examples is that the key findings in the original analysis were stronger in the Honduras example to begin with, with larger differences between quintiles, which provided more room for variation.

There was not a strict correlation between the reduction in the number of variables and in the number of questions. As long as at least one of the response options remains in the list of asset variables, the question needs to be retained. That question, however, may become much more specific. Instead of a question such as “What is the main material of the floor?” followed by all the possible options, the question may become “Is the main material of the floor cement?” that requires a simple yes or no answer. Even though it still counts as a question, obtaining a valid answer to this reformulated question will likely be considerably faster. The drawback, however, is that the development of the questionnaire will require some rephrasing of the original questions, rather than simply removing unnecessary ones.

As noted, we also explored the feasibility of developing a small common set of questions that would be universal and could be used across countries for comparison purposes. Our findings demonstrate that such an approach may be problematic. In our study, which used DHS data from Honduras and Senegal, there was only a 60% overlap in asset variables in the full sets used to construct asset indices. The percentage of overlap dropped in each iteration, indicating that the variables that contribute most to the asset index are to a considerable extent country-specific. This suggests that, depending on the similarities of DHS and other questionnaires, which are usually tailored to reflect country specificities, developing a standardized, simplified asset questionnaire may prove difficult.

The variables that contribute most to the asset index are to a considerable extent country-specific. Developing a standardized, simplified asset questionnaire may therefore prove difficult.

A limiting factor in using a more restricted set of questions is the analysis needed to generate the shortened list of key variables and the associated weights and quintile cut-off points. Much of the process used in this analysis to create such lists can be automated. All the information required for an implementer or researcher to assess the different options of selecting a shorter set of variables and questions and their consequences (i.e., list of variables, questions, and Kappa statistics) and to calculate the asset index for the selected option (i.e., weights, quintile cut-off points, and possibly regression coefficients) could be generated automatically. This would need to be accompanied by additional resources, however, to ensure that an implementer or researcher fully understands the approach. The fact that our analysis showed that there is only limited overlap between the lists of asset variables in the 2 country examples makes such computerization even more relevant, given that a standardized, simplified questionnaire is unlikely to do a good job.

## CONCLUSIONS

It is possible to produce valid asset index results using a limited set of yes/no or multiple choice questions that take only a few minutes to administer to clients. These findings are important. One of the main reasons why many implementers are currently reluctant to assessing whether their intervention is pro-poor is the perceived complexity of the equity analysis involved. Our findings show that this barrier can be overcome. They show that the analysis can be made considerably less burdensome, especially if the simplification process is automated. While additional evidence on the feasibility of the proposed simplification would be welcome, steps can be taken now to make the construction and use of a simplified asset index more user-friendly.

## Notes

**Peer Reviewed**
